# The Mammalian and Yeast A49 and A34 Heterodimers: Homologous but Not the Same

**DOI:** 10.3390/genes12050620

**Published:** 2021-04-22

**Authors:** Rachel McNamar, Katrina Rothblum, Lawrence I. Rothblum

**Affiliations:** Department of Cell Biology, University of Oklahoma Health Sciences Center, Oklahoma City, OK 73104, USA; Rachel-McNamar@ouhsc.edu (R.M.); krothblu@ouhsc.edu (K.R.)

**Keywords:** rRNA transcription, ribosome biogenesis, RNA polymerase I

## Abstract

Ribosomal RNA synthesis is the rate-limiting step in ribosome biogenesis. In eukaryotes, RNA polymerase I (Pol I) is responsible for transcribing the ribosomal DNA genes that reside in the nucleolus. Aberrations in Pol I activity have been linked to the development of multiple cancers and other genetic diseases. Therefore, it is key that we understand the mechanisms of Pol I transcription. Recent studies have demonstrated that there are many differences between Pol I transcription in yeast and mammals. Our goal is to highlight the similarities and differences between the polymerase-associated factors (PAFs) in yeast and mammalian cells. We focus on the PAF heterodimer A49/34 in yeast and PAF53/49 in mammals. Recent studies have demonstrated that while the structures between the yeast and mammalian orthologs are very similar, they may function differently during Pol I transcription, and their patterns of regulation are different.

## 1. Introduction

Ribosome biogenesis is the process that produces the machinery responsible for synthesizing all the proteins in a cell. This process is extraordinarily complex. Ribosome biogenesis requires the coordination of all three nuclear RNA polymerases [1]. RNA polymerase II (Pol II) is responsible for transcribing the genes that encode for the ribosomal proteins and over 200 ribosome assembly factors, including several small nucleolar (sno)RNAs, such as U3 RNA. RNA polymerase III produces the 5S ribosomal RNA (rRNA) and other small nucleolar RNAs that are required for ribosome biogenesis. Small nucleolar RNAs are mainly responsible for guiding rRNA processing and the chemical modification of rRNA, such as methylation and pseudouridylation. RNA polymerase I synthesizes the 47S precursor rRNA in mammals and the 35S precursor in yeast. The 47S precursor is a polycistronic transcript that is cleaved and modified into three separate rRNAs: 18S, 5.8S, and 28S. The rate-limiting step in ribosome biogenesis is rDNA transcription by Pol I [1]. Ribosomal RNA accounts for approximately 80% of the steady-state level of RNA in a cell. Further, ribosome biogenesis is very costly in terms of energy consumption, and can account for up to 70% of the cellular RNA synthesis in proliferating cells [1,2]. A proper balance of ribosomes is required to maintain cellular homeostasis and support cell growth. Therefore, it is key that ribosome biogenesis be heavily regulated to help maintain cellular homeostasis and prevent the onset of disease. The dysregulation of ribosome production contributes to many pathologies, such as cancer, cardiac hypertrophy, and aging, as well as developmental disorders like ribosomopathies [3,4,5].

Earlier dogma stated that the rate of Pol I transcription in cells was constant, and there was no regulation of this process. Over many years, research has shown that many pathways that regulate cell growth also play roles in regulating rDNA transcription. Both oncogenes and anti-oncogenes, such as c-Myc and Rb, contribute to the regulation of transcription by Pol I [6,7,8]. Furthermore, rDNA transcription is regulated via post-translational modifications of either core subunits of the polymerase or Pol I transcription factors [9,10,11,12]. Similar to the Pol II system, transcription factors are required for promoter recognition, preinitiation complex (PIC) formation, and progression through the transcription cycle (initiation, elongation, and termination). Pol I transcription factors can be separated into two groups: general transcription factors and polymerase-associated factors (PAFs). In mammals, the general transcription factors include a multi-subunit complex, referred to as SL1 (selectivity factor 1) and UBF (upstream binding factor) [13]. SL1 promotes Pol I PIC formation by binding to both the core promoter and upstream promoter elements in the rDNA repeat; with UBF, it forms a stable interaction with the rDNA promoter to produce the committed template. In vivo, SL1 and UBF act together to recruit Pol I to the rDNA and promote efficient PIC formation. In vitro, SL1 is sufficient to direct accurate transcription initiation. In yeast, there are also two general transcription factors: core factor (CF) and UAF (upstream activating factor) [14]. Both of these consist of several subunits. The core factor is the yeast counterpart of SL1 and functions similarly during promoter recognition, PIC formation, and transcription initiation. UAF is a multimeric protein complex that is unique to the yeast system. The second group of identified Pol I transcription factors in mammals, the PAFs, include three dissociable proteins: PAF53, PAF49, and Rrn3 (also known as TIF-1A). In yeast, the orthologs of PAF53 and PAF49 are A49 and A34, respectively. The yeast homolog of Rrn3 is yeast Rrn3. The goal of this review is to compare and contrast how the mammalian PAF53/49 heterodimer and the yeast A49/34 heterodimer function during rDNA transcription. Further, we will consider mechanisms that regulate the activities of the heterodimers, and how this regulation affects Pol I transcription.

## 2. The Rpa49 and Rpa34 Protein Families: Mammalian vs. Yeast Heterodimers

In 1975, Fromageot’s lab identified two forms of yeast RNA polymerase I [15]. The difference between them was the presence or absence of two polypeptide chains: Rpa49 and Rpa34. These two proteins are the yeast orthologues of PAF53 and PAF49, respectively. The dissociation of these two proteins from the polymerase caused a reduction in its ability to transcribe rDNA, but had no effect on its general transcription activity. Similarly, multiple labs have shown that mammalian RNA polymerase I is also present in two forms [16,17,18]. The difference between these two forms has been ascribed to the presence or absence of the “third-largest polypeptide” of Pol I. Pol IB, containing a putative “third” polypeptide, is able to support both specific and random transcription, while Pol IA, missing the “third” polypeptide, is only able to support random transcription in vitro and was not active in vivo. In 1996, Muramatsu’s laboratory [19] showed that the “third-largest polypeptide” of mammalian/mouse Pol I could be resolved into three polypeptides: PAF53, PAF51, and PAF49 (named for their molecular masses). Results from this study concluded that PAF53 and PAF51 are related, while PAF53 and PAF49 are two distinct, unrelated proteins. This led to future studies that focused on PAF53 and PAF49. A few years later, Muramatsu’s lab was able to demonstrate that PAF53 and 49 form a heterodimer [20]. Beckouet et al. also showed that Rpa49 and Rpa34 heterodimerize in vivo [21]. These studies served as a platform that promoted further research into the roles these dissociable factors play in facilitating rDNA transcription. While the yeast and mammalian factors were discovered around the same time, studies on transcription by Pol I were greatly facilitated by the ability to combine genetics and biochemistry using yeast. Our goal for this section is to highlight the similarities and differences between the yeast heterodimer and the mammalian heterodimer, in order to identify the gaps in knowledge that still need to be explored for mammalian PAFs.

### 2.1. Structure and Sequence

**Figure 1:**
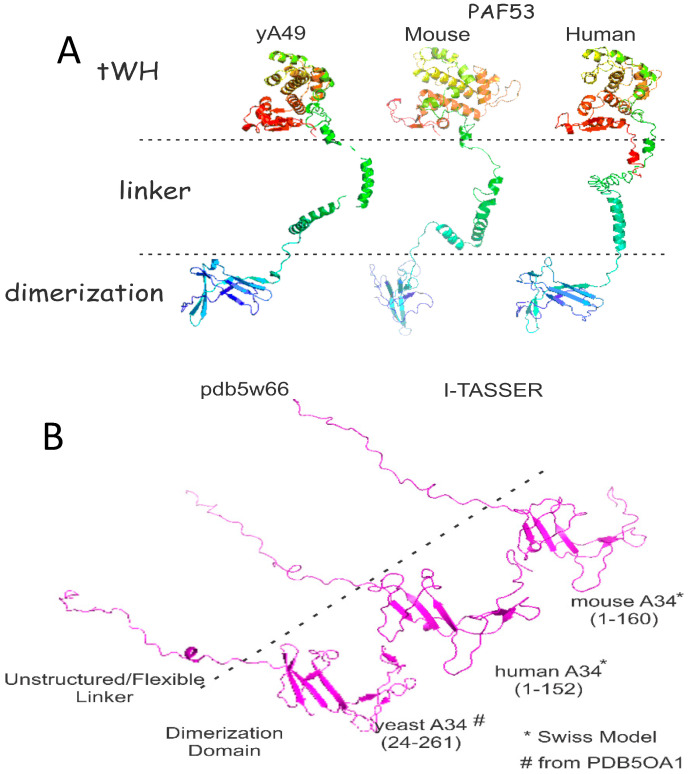
Predicted structural similarities of the A49 orthologs (**A**) and the A34 orthologs (**B**).

**Figure 2:**
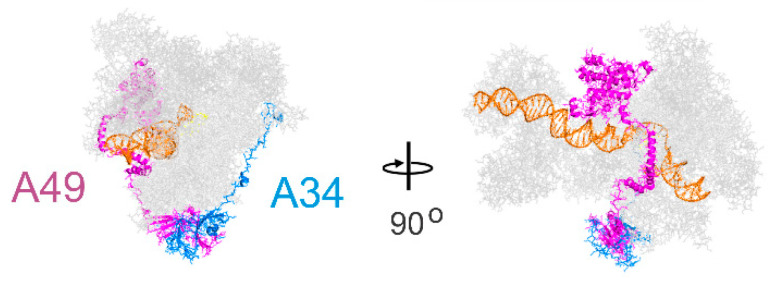
Placement of the RPA34 (blue)–RPA49 (magenta) heterodimer in RNA polymerase I. The DNA is orange, and the remainder of the polymerase subunits are grey. Based on PDB5W66.

**Figure 3:**
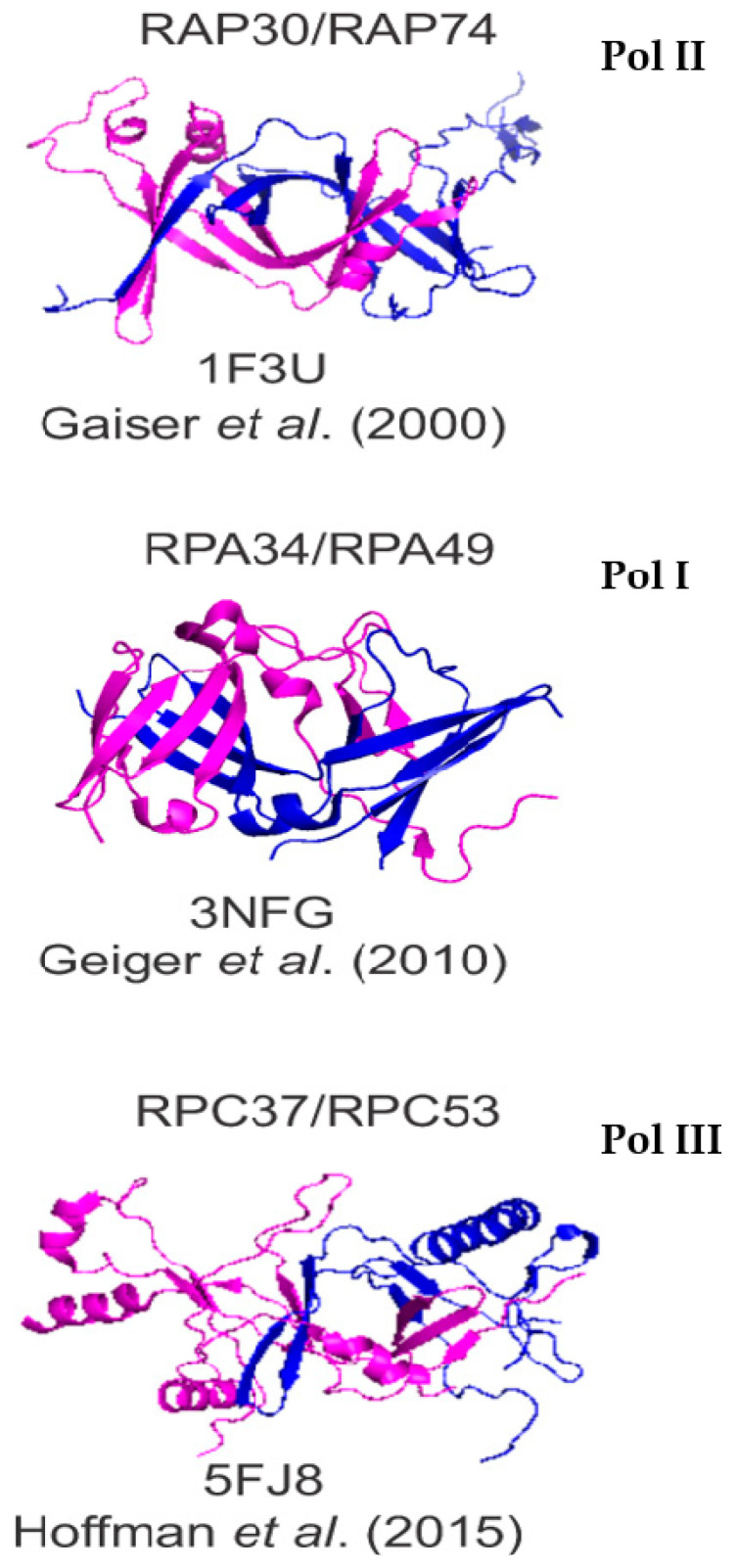
Structural similarities of the dimerization modules of the homologous subunits of Pol II (RAP30/74), Pol I (A49/A34), and Pol III (RPC37/53) [24,25,26].

The sequences of the yeast and mammalian orthologues do not demonstrate significant identities. However, focusing on A49/PAF53 and A34/PAF49, the structures predicted for the mammalian proteins are strikingly similar to those of the yeast protein. This is illustrated in Figure 1 for the yeast, human, and mouse orthologues of A49 and A34 (upper and lower panels, respectively). The predicted mammalian structures were derived by I-Tasser or Swiss Model, as indicated [22,23]. The heterodimers bind to the lobe of Pol I (Figure 2). Strikingly, the dimerization domains of A49/A34 are similar to the triple barrel folds characteristic of the dimerization domains of the Pol II transcription factor TFIIF (Rap74/Tfg1 and Rap30/Tfg2) and the Pol III heterodimer of C37 and C53 (Figure 3 [24,25,26]). In yeast, TFIIF is composed of three subunits: Tfg1, Tfg2, and Tfg3. Tfg1 and Tfg2 are the counterparts of the human TFIIF subunits Rap74 and Rap30, respectively [27]. Interactions between Rap74 and Rap30 form the dimerization domain of TFIIF. C37 and C53 are two subunits of RNA Polymerase III that form a heterodimeric complex similar to the A49/34 and RAP74/30 heterodimers of Pol I and Pol II, respectively [24]. Moreover, the dimerization domains of the orthologues are found at the N-termini of the yeast and mammalian proteins, and the predicted structures of the mammalian dimerization domains are similar to those of the deduced yeast proteins [28]. Although the predicted structures of the yeast and mammalian orthologues are similar, they did not form heterodimers across species, indicating sequence-specific protein–protein interactions [28]. Interestingly, the TFIIF, A49/A34, and C37/C53 heterodimers all bind to the lobes of their respective polymerases, as shown in Figure 2. Furthermore, the relatively unstructured arm of A34/PAF49 extends along the same side of the polymerase as the extended flexible linker of TFIIF in recent cryoEMs (see Figure 2 in [29]).

**Figure 4:**
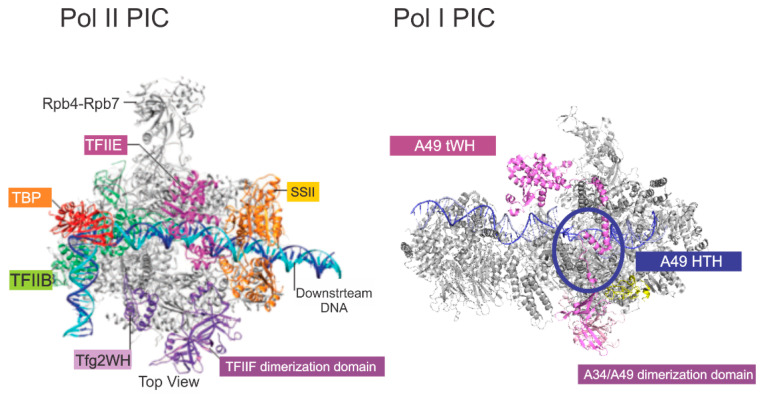
Comparison of the PICs formed by RNA polymerases II and I. The Pol II PIC is from [39], and the Pol I PIC is from PDB5w66.

TFIIF has multiple roles in Pol II transcription. It has been reported to reduce non-specific interaction of Pol II with DNA [30] and stabilizes the PIC [31]. TFIIF also affects the start site selection, stimulates initial RNA synthesis, stabilizes the transcription bubble, and reduces pausing [32,33,34,35,36,37]. Similarly, Geiger et al. [24] reported that the yeast A49/A34 dimerization domain stimulates RNA cleavage activity. They also demonstrated that a tandem winged helix (tWH) found in the C-terminal domain of A49 had DNA-binding activity and increased the processivity of Pol I, both of which are properties of TFIIE. In their analysis of the domains of PAF53, McNamar et al. [38] found that the predicted tWH of mammalian PAF53 also had DNA-binding activity, and that this domain was essential for full activity. They also found that both the dimerization domain and the linker region were essential for full activity. Analysis of the linker demonstrated the presence of a helix–turn–helix (HTH). Further experiments demonstrated that this HTH was a second DNA-binding domain. The HTH was found to be essential for PAF53 activity, and an analogous domain in yeast A49 was also essential for cell growth. Both the tWH and the HTH may function in orienting the polymerase around the transcription start site (Figure 4). The HTH of PAF53 may interact with the transcribed DNA in a position analogous to that of the winged helix of TFg2 [39].

Interestingly, the tWH domains of TFIIE and A49 both lie upstream of the transcription initiation site, and the linker domains of TFIIF and TFIIE appear to span the polymerase cleft in a manner similar to the HTH of A49. In Pol I, the structure of the tWH and linker domains of A49 appear to be more dynamic than those of TFIIE, as they were not visible in the majority of the cryo-EM images captured in the past [40,41,42,43,44].

### 2.2. The PAFs’ Function as a Heterodimer

A34/PAF49 and A49/PAF53 form a heterodimer complex in both in vitro and in vivo settings. It has also been hypothesized that these proteins function as a heterodimer. In Fromageot’s 1975 paper, they demonstrated that upon purification of RNA polymerase I, there were two forms: Pol IA and Pol IB [15]. The difference between these two forms was the presence or absence of the heterodimer. The paper did not address whether either one of the components of the heterodimer could interact with polymerase in the absence of its partner. Liljelund et al. observed that a complete deletion of A49 from yeast did not cause A34 to dissociate from the polymerase [45]. Alternatively, ChIP assays performed by Beckouet et al. demonstrated that when the N-terminal domain of A49 was deleted, A34 was completely lost from the rDNA bound polymerase [21]. Further, Gadal et al. found that when A34 was deleted, purified RNA polymerase I lacked the A49 subunit [46]. This observation contradicted the results from their growth assays they performed on both A49-Δ and A34-Δ yeast strains. The *S. cerevisiae* A49-Δ strain grew slowly at 30 °C and not at all when grown at 25 °C. In contrast, the *S. cerevisiae* A34-Δ strain grows at normal rates at either temperature. These observations imply that A49 can be incorporated into the core polymerase in the absence of A34. The production of a polymerase devoid of A49 when A34 is deleted demonstrates that the polymerase has a decreased stability in vitro. Thus, A34 helps stabilize the association of A49 with Pol I. Studies in yeast have demonstrated that A49 can be recruited to the polymerase independently of A34. For example, the deletion of the heterodimerization domain does not affect proliferation [21,38]. However, deletion of the dimerization domain of PAF53 significantly inhibited proliferation [38]. This may be due to differences in the interactions between the orthologs and core Pol I in yeast and mammals.

Until recently, it has been difficult to perform similar studies on the mammalian RNA polymerase I system, due to an inability to knock out PAF53 and/or PAF49. A CRISPR study performed by our lab discovered these difficulties [47]. When PAF53 was CRISPRed to knock out the gene, the cells would undergo recombination in order to continue expressing a functional PAF53 protein. Knock out of the endogenous gene was only possible if wild-type PAF53 was ectopically expressed while the cells were being CRISPRed. Therefore, most previous studies that investigated the functional relationship between PAF53 and PAF49 were performed via overexpression experiments. Penrod et al. demonstrated that when PAF53 and PAF49 were both ectopically expressed, there was an increase in expression levels of both proteins when compared to their levels when each was expressed individually [9]. They also demonstrated that both overexpressed PAF53 and PAF49 interacted with endogenous RNA polymerase I. Recently, our lab has developed a system that allows us to induce and target the degradation of endogenous PAF53 and PAF49 in HEK293 cells [38,48,49]. Generally, the system allows us to quickly degrade our target protein. After we induce degradation, we are able to study the effects knockdown of our target protein has on the composition and function of Pol I, as well as effects on cellular physiology. With this system, we will be able to perform future studies, in order to determine if PAF49 can associate with the polymerase in the absence of PAF53 and vice versa. While we know that both components of the yeast heterodimer can bind to the polymerase in the absence of its partner, it is still unclear whether PAF49 and PAF53 can bind to the polymerase independently of each other. Furthermore, based on observations in yeast that deletion of A34 has no effect on growth rates, the components of the heterodimer may be able to function individually.

### 2.3. Are PAFs Necessary for rDNA Transcription? Cell Growth? Cell Viability?

A long-standing question in the field of RNA polymerase I transcription is the necessity of the heterodimer. That is, are A34/PAF49 and/or A49/PAF53 essential for rDNA transcription, cell proliferation, or cell viability? Many studies performed with *S. cerevisiae* report that the A49/34 heterodimer is not essential for cell proliferation or cell viability [21,45,46]. This may be due to operational definitions. Studies done in A49-Δ yeast strains show that these strains grow slowly at 30 °C and fail to grow at temperatures of 25 °C and 37 °C. Furthermore, the ΔA49 mutation causes sensitivity to mycophenolate, a drug that causes the depletion of guanosine nucleotides, and lethality in cells lacking the nonessential A14 polymerase subunit or the HMG box protein Hmo1. Interestingly, RPA51, the functional homolog of A49 found in *S. pombe* (fission yeast), is also required for wild-type levels of cell growth [50]. When Nakagawa et al. knocked out RPA51, they also found that the yeast grew slowly at 30 °C and failed to grow at lower temperatures. Transcription analysis of Pol I isolated from RPA51 null yeast demonstrated that it wasn’t essential for non-specific transcription, but was required for specific transcription. Fromageot et al. had also demonstrated that Pol I, devoid of the A49/A34 heterodimer, was capable of non-specific transcription [15]. Therefore, while A49 is technically not essential for rDNA transcription, viability, and cell growth, it is required for specific transcription and for normal levels of cell proliferation. In this case, non-specific transcription refers to the polymerase’s ability to synthesize a strand of RNA from a random sequence of DNA. On the other hand, specific transcription refers to the polymerase’s ability to initiate transcription from the rDNA promoter. Therefore, while the enzymatic activity of Pol I remains unperturbed in the absence of A49, incorporation of A49 into the Pol I holoenzyme is required to promote transcription at the rDNA promoter. In contrast to the A49 null mutants, A34-Δ yeast strains grow at wild-type rates and have little to no effect on levels of rDNA transcription [46]. These results demonstrate that A34 is not required for both non-specific and specific transcription.

Muramatsu’s lab was the first to report that PAF53 and PAF49 are required for specific transcription [19,20]. The results from his lab showed that antibodies to these two proteins blocked specific transcription of mouse rDNA in vitro, but had no effect on non-specific transcription. Transcription was rescued when the recombinant protein was added to the in vitro transcription reaction. Furthermore, the authors performed in vivo primer extension assays to determine if deleting the N- or C-terminus of PAF49 had any effect on transcription. Results from these experiments showed that overexpression of the C-terminus deletion mutant in a wild-type background caused a decrease in pre-rRNA synthesis. While deletion of the N-terminus had no effect, the results from the in vivo experiment suggest that the C-terminus deletion mutant might be having a dominant–negative effect. While the conclusions from these papers had great implications for the role(s) PAF53 and PAF49 play during rDNA transcription, there are some limitations to the assays they used that warrant further investigation. First, transcription could have been blocked due to steric hindrance, not due to the inhibition of the activity of the PAF. Second, the in vivo primer extension assays were performed in a wild-type background instead of in a PAF49-null background. It is hard to make solid conclusions about PAF49’s importance during transcription when mutant proteins are being assessed in the presence of the full-length protein. Our lab used these experiments as a jumping off point to further investigate the roles PAF53/49 plays during rRNA synthesis.

**Figure 5:**
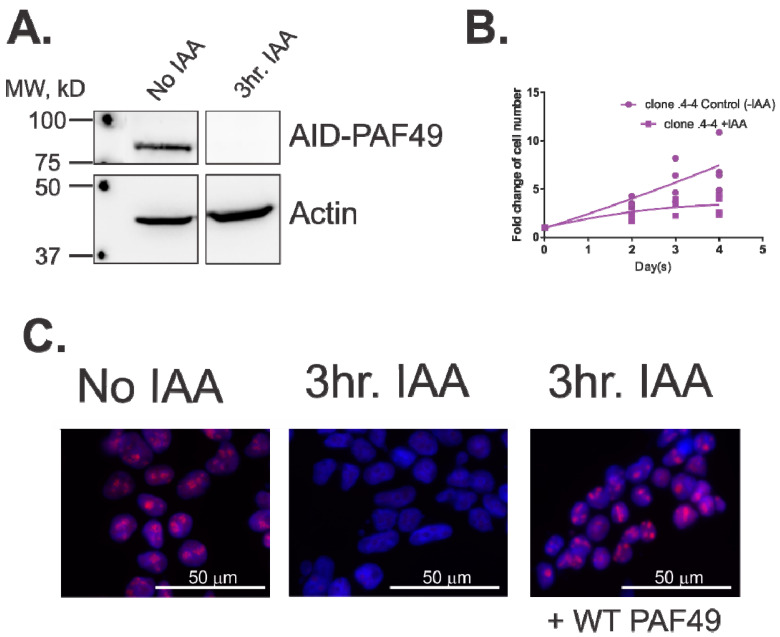
PAF49 is required for cell proliferation and rDNA transcription. (**A**) IAA induces the rapid degradation of AID-PAF49. The endogenous PAF49 gene in HEK293 cells was tagged with an auxin-inducible degron (AID) [38]. Cells were treated with or without indole-3 acetic acid (IAA) for 3 h. Whole-cell extracts were prepared, and levels of PAF49 were determined via Western blot analysis. (**B**) PAF49 is required for proliferation. Cells containing AID-PAF49 were treated with or without IAA for 4 days. On days 0, 2, 3, and 4, live cells were counted as described [38]. (**C**) PAF49 is required for pre-rRNA synthesis. After cells were treated with IAA for three hours, they were pulsed with 5-ethyl uridine (EU) for 15 min, and de novo synthesized RNA was visualized as described (red) against a DAPI background [51]. To rescue rDNA transcription, wild-type mouse PAF49 (WT PAF49) was ectopically expressed for 24 h before the cells were treated with IAA.

To determine if PAF53 or PAF49 are essential for rDNA transcription, our lab utilized an auxin-inducible degron system, in which the endogenous PAF could be targeted for rapid degradation in <1 h [38,48,49]. We chose this system because previous experiments performed by our lab had demonstrated that PAF53 could not be knocked out via CRISPR, in agreement with genome-wide CRISPR knock out studies [47]. Further, siRNAs for the PAFs require days of treatment to completely knock down the proteins. Since knockdown via siRNAs is slow, the cell could compensate for the loss of the protein, and our results would be skewed because of the compensation. Therefore, we used an auxin-inducible degron system to target each PAF individually, in order to determine if they were essential for rDNA transcription, cell proliferation, and cell viability. We demonstrated that PAF53 is essential for rDNA transcription and cell proliferation [38]. Alternatively, knockdown of PAF53 was not essential for cell viability—i.e., the cells ceased to proliferate, but did not die for at least seven days. We also demonstrated that ectopic expression of wild-type PAF53 could rescue proliferation and transcription. Current studies in the lab are focusing on the role PAF49 plays during rDNA transcription. As shown in Figure 5, we demonstrate that when PAF49 is knocked down (Figure 5A), rDNA transcription (Figure 5C) and cell proliferation (Figure 5B) is inhibited. On the other hand, knock down of PAF49 has no effect on viability (Figure 5B). In Figure 5B, cells were counted via the trypan blue exclusion assay. It was demonstrated that while the KD of PAF49 inhibits cell proliferation, there is no decrease in total cell number. Additionally, there was no difference in the percentage of live cells between the control and the PAF49 KD cells. Furthermore, ectopic expression of wild-type PAF49 rescues rRNA synthesis (Panel C). The results from our studies confirm Muramatsu’s conclusions that PAFs are essential for specific rDNA transcription.

The overall conclusion that both PAF49 and PAF53 are essential for rDNA transcription, and that cell proliferation shifts the paradigm that results from experiments investigating RNA polymerase I transcription in yeast could be used to understand Pol I transcription in mammals (i.e., humans). Our demonstration that PAF49 is essential for growth and transcription while its ortholog, yeast A34, is nonessential, highlights the importance of further studying Pol I transcription in mammals.

### 2.4. What Roles Do PAFs Play during rDNA Transcription?

As mentioned above, both the yeast and mammalian heterodimers play an essential role in facilitating rRNA synthesis. Specifically, in yeast A49 is required for specific transcription, while A34 seems to be dispensable. Alternatively, both PAF53 and PAF49 are essential to promote rDNA transcription in mammals. A major question in the field of Pol I transcription is how do the PAFs help facilitate specific rDNA transcription?

Currently, the majority of studies focusing on the function of the PAFs have been performed in yeast. These have focused on the main steps in transcription: initiation, elongation, and termination.

#### 2.4.1. Promoter Recognition and Initiation

Just like in the Pol II and Pol III systems, Pol I relies on general transcription factors, which help it bind to the promoter and form a transcription-competent initiation complex. In yeast, those GTFs include a core factor (CF) and upstream activation factor (UAF). In mammals, the GTFs are selectivity factor-1 (SL-1) and upstream binding factor (UBF). In both the yeast and mammalian systems, the TATA-binding protein (TBP) in CF and SL-1 are essential for recruiting Pol I to the rDNA promoter. Before Pol I binds to the promoter, it must first form a complex with Rrn3, a polymerase-associated factor that is released once transcription has been initiated [52]. It has been hypothesized that the heterodimer helps facilitate interactions between the GTFs and Rrn3 with the core polymerase. Muramatsu’s lab demonstrated that PAF53 interacts with UBF and PAF49 interacts with TAF_I_48, a subunit of SL-1 [19,20]. Multiple cryo-EM studies that have investigated the mechanisms of RNA polymerase I promoter recognition and initiation have shown that the tWH domain of A49 may play a role in priming Pol I for promoter escape by displacing CF and Rrn3 from Pol I [42,53,54]. Previously, Beckouet et al. had reported that deletion of A49 impaired the recruitment of Rrn3 to the rDNA promoter and significantly reduced the release of Rrn3 from the polymerase [21]. Furthermore, many of the cryo-EM structures of A49 bound to the polymerase fail to show the full-length protein. This could be due to the dynamic nature of A49, since multiple studies have shown it to play roles in both initiation and elongation. Our lab has reported that PAF53 has two DNA-binding regions, one in the tWH domain and one in the linker region [38]. Cramer’s lab has also shown that the tWH domain of A49 has DNA-binding activity [24]. Upon further investigation, McNamar et al. reported that the linker region of PAF53 and A49 was necessary for growth [38]. Our current model shows that the linker region of PAF53/A49 binds to the DNA in the cleft in order to help melt and stabilize the DNA. While the linker spans the cleft, conformational changes in the C-terminal end of PAF53/A49 position the tWH so that it can help the polymerase switch from its open initiation complex to its elongation complex.

#### 2.4.2. Elongation

RNA polymerase I processivity during elongation plays a key role in successfully synthesizing a complete rRNA gene. Similarly to Pol II and Pol III, Pol I recruits elongation factors that help it remain on the DNA until a termination signal is reached [55]. Studies performed with *S. cerevisae* have demonstrated that the heterodimer act as built-in Pol I elongation factors. Cramer’s lab reported that A49/34 is required for normal elongation activity of Pol I in vitro [24,56]. ΔA34 yeast strains were sensitive to 6-azauracil (6AU) and showed a slow-growth phenotype. This demonstrates that when the nucleotide supply was limited by 6AU, Pol I devoid of A34 could not properly elongate. These results suggest that the heterodimer is required for normal RNA elongation by Pol I in vitro and in vivo. Specifically, the tWH domain of A49 was found to be required for processivity. Additional studies need to be performed in mammalian cells to determine the roles PAF53/49 play during elongation. Since there are differences between the yeast and mammalian heterodimer, we can only speculate that the mammalian PAFs play similar roles in Pol I elongation.

#### 2.4.3. Termination

In both yeast and mammals, there are termination factors that bind to the 3’ end of the rDNA gene [57,58]. During termination, the polymerase dissociates from the DNA and releases the synthesized rRNA strand. It has been hypothesized that the cleavage activity of Pol I may be involved during termination. Immediately following termination, approximately 10 nucleotides are cleaved from the 3’ end of the pre-rRNA [59]. Intrinsic RNA cleavage activity could be required for this rRNA trimming event. Kuhn et al. reported that a complete polymerase cleaved RNA more efficiently than a polymerase devoid of the heterodimer [56]. A follow-up study by Geiger et al. demonstrated that normal RNA cleavage activity requires a dimerization module between A49/A34 and either the linker of A49 or the C-terminal tail of A34 [24]. These results suggest that the dimerization module of the heterodimer is required for full RNA cleavage activity—i.e., the heterodimer must be associated with Pol I. More studies need to be performed with the yeast and mammalian Pol I system to fully understand what role the heterodimer plays during the termination of Pol I transcription.

### 2.5. Regulation of the PAFs

It has been reported that there is post-transcriptional regulation of the mammalian PAFs at several levels. In their initial reports of PAF53 and PAF49, Muramatsu’s laboratory reported that they could identify two populations of Pol I, one that contained the PAF53/49 heterodimer and one that did not [19,20]. Hannan et al. reported that the molar ratio of PAF53 to A127 in affinity-purified Pol I was 0.6, confirming that only some of the Pol I complexes contained PAF53 [60]. Muramatsu’s laboratory also reported that upon serum starvation, the two proteins translocated from the nucleoli of NIH 3T3 cells [19,20]. In contrast, Seither et al. reported the “constitutive and strong association of PAF53 with RNA polymerase I” [61], and argued that it “is not a loosely associated regulatory factor but a bona fide subunit of Pol I.” Further evidence in support of the model for the regulation of the level of PAF53 in the cell was reported by Hannan et al. when they found that insulin stimulated rDNA transcription in serum-starved 3T6 cells and exponentially growing H4–E–C3 cells [62]. Moreover, they reported that PAF53 levels increased concomitantly with rDNA transcription. Interestingly, they reported that serum starvation caused an 80% reduction in the level of PAF53 in the NIH3T6 cells, not just a translocation from the nucleolus. The apparent discrepancy as to whether serum affected PAF53 levels in exponentially growing cells was examined by Penrod et al. [9]. They found that serum deprivation of NIH 3T6 cells resulted in a 70% decrease in the whole cell levels of PAF53 and PAF49. However, this same decrease was not found in NIH 3T3, HEK 293, or CHO cells. This series of observations led to a quandary. Were the steady-state levels of PAF53/PAF49 subject to regulation? To address this question, Penrod et al. examined the nuclear distribution of PAF53 and PAF49 in NIH 3T3 cells, a cell line that did not demonstrate a dramatic change in steady-state levels of the proteins. Their results confirmed those of Yamamato et al., which were that serum starvation caused a delocalization of PAF53 and PAF49 from the nucleoli [19,20]. Further, biochemical fractionation of Pol I from those cells demonstrated the dissociation of PAF53 from core polymerase [9]. These observations raise two questions: what regulates the association of PAF53 and PAF49 with Pol I, and does the dissociation of the heterodimer from Pol I regulate rDNA transcription?

**Figure 6:**
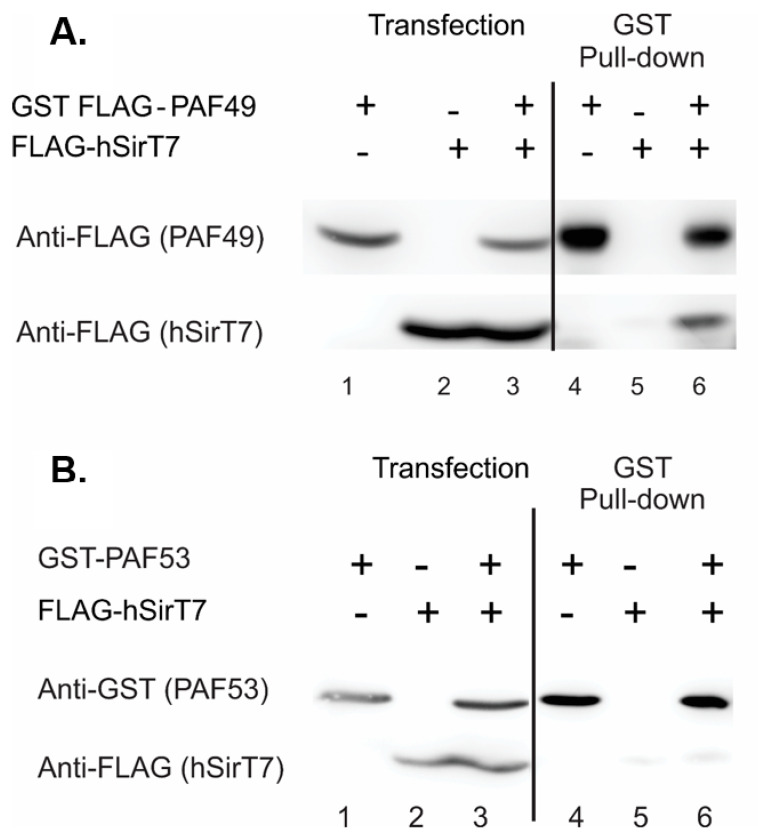
SIRT7 interacts with PAF49 (**A**), but not with PAF53 (**B**). HEK293 cells were transfected with vectors expressing the indicated proteins. Forty-eight hours following transfection, cell lysates were prepared, and the ectopically expressed proteins subjected to affinity purification (GSH). The input (10%) and the proteins that bind to the affinity matrix were analyzed by SDS-PAGE and Western blotting, as described [9].

The second question is significant, because studies in yeast suggest that while the heterodimer may play significant roles in rDNA transcription, it is not essential [21,24,45,46,50,63]. However, genetic screens of the human genome have suggested that both proteins are “essential” for mammalian cell proliferation [64,65,66]. Our initial attempt to obviate PAF53 expression using CRISPR/Cas9 [47] has demonstrated that the protein was essential. Finally, a series of experiments in which the endogenous PAF53 gene was modified to include an inducible degron has demonstrated the essential role for PAF53 in rDNA transcription and cell proliferation [38]. What regulates the activity of the PAF53/PAF49 heterodimer? The post-translational regulation of the heterodimer is unknown. The pathway(s) that leads to the downregulation of steady-state levels or to the dissociation of the heterodimer from Pol I is not known. Panov et al. reported evidence for growth-regulated tyrosine phosphorylation of PAF49 (CAST) in initiation-competent Pol I [67]. There are several reports that PAF53 and PAF49 are acetylated [9,68,69], and the sir 2 analog, SIRT7, has been implicated in the control of rDNA transcription [69,70,71,72,73]. We found that PAF49 was acetylated. Acetylation did not affect heterodimerization, but the hypoacetylated forms of PAF49 more strongly interacted with core Pol I [9]. We also found that while SIRT7 bound to PAF49 (Figure 6A), it did not bind to PAF53 (Figure 6b). Again, further studies are needed to determine the specific sites of acetylation, the roles of acetylation in modulating the functions of the heterodimer, and the enzymes (pathways) that are involved in acetylation and deacetylation. Along these lines, it should be noted that SIRT1 and SIRT6 are also found in the nucleolus, as are various lysine acetyl-transferases.

**Figure 7:**
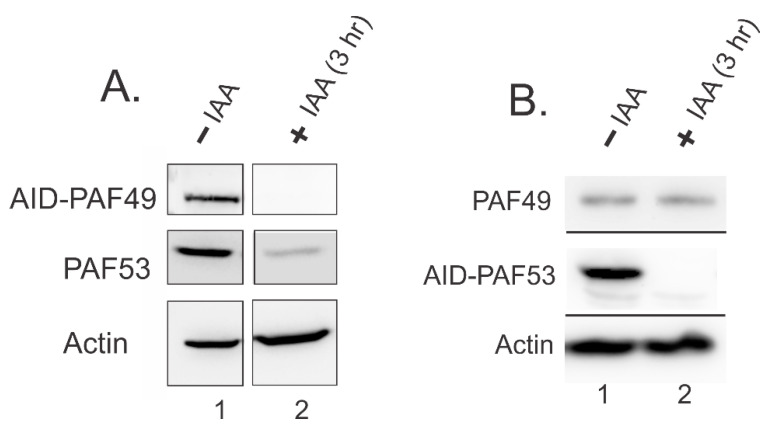
Targeted degradation of PAF49 results in the rapid depletion of PAF53 (**A**), but not vice versa (**B**). HEK293 cells expressing either AID-PAF49 or AID-PAF53 were treated with IAA for 3 h. After that, whole cell lysates were prepared, and the levels of PAF49 and PAF53 were investigated via Western blot analysis. (**A**) Lane 2 shows that upon targeted degradation of PAF49, the levels of PAF53 were depleted. (**B**) Lane 2 shows that targeted degradation of PAF53 did not cause the rapid depletion of PAF49.

Penrod et al. found that ectopic co-expression of PAF53 and PAF49 in HEK293 caused an increased accumulation of both proteins when compared to ectopic expression of each protein individually [9]. This led to the hypothesis that heterodimerization of PAF53/49 regulates the stability of the two proteins in vivo. Recently, our lab investigated whether knockdown of one protein in the heterodimer causes an effect on the levels of the remaining protein. In Figure 7, we demonstrate that auxin-induced degradation of PAF49 caused the rapid degradation of PAF53 (Panel A). Alternatively, knockdown of PAF53 via the same auxin-inducible degron system did not cause the rapid degradation of PAF49 (Panel B). Currently, we hypothesize that PAF49 is more stable without its partner, because it is able to interact with polymerase by itself. This interaction may cause PAF49 to be stable for longer periods of time. On the other hand, PAF53 needs to be dimerized with PAF49, in order to be able to interact with Pol I. If PAF53 is not able to incorporate into the polymerase, it might become unstable and be targeted for degradation. Interestingly, studies in yeast have not reported that the knockout of A34 causes the knockdown of A49. It would be interesting to see if the knockout of A34 caused an effect on the protein levels of A49, and vice versa. Future studies need to be performed to test our current model. Additionally, further studies are needed to determine how PAF49 and PAF53 are co-regulated, and how they regulate their partner.

## 3. Conclusions

While there are similarities between the yeast and mammalian Pol I systems, it is important that we understand the differences between yeast and mammalian Pol I transcription. Understanding these differences will aid in future studies targeting Pol I transcription in different disease models. In this review, we highlighted some key differences between the yeast and mammalian PAF heterodimers. Importantly, we demonstrated that while A34 is completely dispensable in yeast Pol I transcription, PAF49, its ortholog, is required for mammalian Pol I transcription. We also highlighted the differences between yeast A49 and mammalian PAF53. Specifically, the tWH of A49 is sufficient to rescue rDNA transcription in yeast, but the tWH of PAF53 does not rescue transcription in mammals. This infers that recruitment of the heterodimer may differ between yeast and mammals. Lastly, we have observed that levels of PAF49 and PAF53 in the cell are co-regulated, while there is no evidence that demonstrates the same regulation between A34 and A49.

## Data Availability

Data available on request.

## References

[1] Warner J.R. (1999). The economics of ribosome biosynthesis in yeast. Trends Biochem. Sci..

[2] Warner J.R., Vilardell J., Sohn J.H. (2001). Economics of ribosome biosynthesis. Cold Spring Harb. Symp. Quant. Biol..

[3] Hannan K.M., Sanij E., Rothblum L.I., Hannan R.D., Pearson R.B. (2013). Dysregulation of RNA polymerase I transcription during disease. Biochim. Et. Biophys. Acta.

[4] Danilova N., Gazda H.T. (2015). Ribosomopathies: How a common root can cause a tree of pathologies. Dis. Model. Mech..

[5] Tiku V., Antebi A. (2018). Nucleolar Function in Lifespan Regulation. Trends Cell Biol..

[6] Hannan K.M., Hannan R.D., Smith S.D., Jefferson L.S., Lun M., Rothblum L.I. (2000). Rb and p130 regulate RNA polymerase I transcription: Rb disrupts the interaction between UBF and SL-1. Oncogene.

[7] Hannan K.M., Kennedy B.K., Cavanaugh A.H., Hannan R.D., Hirschler-Laszkiewicz I., Jefferson L.S., Rothblum L.I. (2000). RNA polymerase I transcription in confluent cells: Rb downregulates rDNA transcription during confluence-induced cell cycle arrest. Oncogene.

[8] Arabi A., Wu S., Ridderstrale K., Bierhoff H., Shiue C., Fatyol K., Fahlen S., Hydbring P., Soderberg O., Grummt I. (2005). c-Myc associates with ribosomal DNA and activates RNA polymerase I transcription. Nat. Cell Biol..

[9] Penrod Y., Rothblum K., Cavanaugh A., Rothblum L.I. (2014). Regulation of the association of the PAF53/PAF49 heterodimer with RNA polymerase I. Gene.

[10] Cavanaugh A.H., Hirschler-Laszkiewicz I., Hu Q., Dundr M., Smink T., Misteli T., Rothblum L.I. (2002). Rrn3 phosphorylation is a regulatory checkpoint for ribosome biogenesis. J. Biol. Chem..

[11] Fath S., Milkereit P., Peyroche G., Riva M., Carles C., Tschochner H. (2001). Differential roles of phosphorylation in the formation of transcriptional active RNA polymerase I. Proc. Natl. Acad. Sci. USA.

[12] Blank M.F., Chen S., Poetz F., Schnolzer M., Voit R., Grummt I. (2017). SIRT7-dependent deacetylation of CDK9 activates RNA polymerase II transcription. Nucleic Acids Res..

[13] Russell J., Zomerdijk J.C. (2006). The RNA polymerase I transcription machinery. Biochem. Soc. Symp..

[14] Aprikian P., Moorefield B., Reeder R.H. (2001). New model for the yeast RNA polymerase I transcription cycle. Mol. Cell. Biol..

[15] Huet J., Buhler J.M., Sentenac A., Fromageot P. (1975). Dissociation of two polypeptide chains from yeast RNA polymerase A. Proc. Natl. Acad. Sci. USA.

[16] Tower J., Sollner-Webb B. (1987). Transcription of mouse rDNA is regulated by an activated subform of RNA polymerase I. Cell.

[17] Schwartz L.B., Roeder R.G. (1974). Purification and subunit structure of deoxyribonucleic acid-dependent ribonucleic acid polymerase I from the mouse myeloma, MOPC 315. J. Biol. Chem..

[18] Matsui T., Onishi T., Muramatsu M. (1976). Nucleolar DNA-dependent RNA polymerase from rat liver. 2. Two forms and their physiological significance. Eur. J. Biochem..

[19] Hanada K., Song C.Z., Yamamoto K., Yano K., Maeda Y., Yamaguchi K., Muramatsu M. (1996). RNA polymerase I associated factor 53 binds to the nucleolar transcription factor UBF and functions in specific rDNA transcription. EMBO J..

[20] Yamamoto K., Yamamoto M., Hanada K., Nogi Y., Matsuyama T., Muramatsu M. (2004). Multiple protein-protein interactions by RNA polymerase I-associated factor PAF49 and role of PAF49 in rRNA transcription. Mol. Cell. Biol..

[21] Beckouet F., Labarre-Mariotte S., Albert B., Imazawa Y., Werner M., Gadal O., Nogi Y., Thuriaux P. (2008). Two RNA polymerase I subunits control the binding and release of Rrn3 during transcription. Mol. Cell. Biol..

[22] Waterhouse A., Bertoni M., Bienert S., Studer G., Tauriello G., Gumienny R., Heer F.T., de Beer T.A.P., Rempfer C., Bordoli L. (2018). SWISS-MODEL: Homology modelling of protein structures and complexes. Nucleic Acids Res..

[23] Yang J., Yan R., Roy A., Xu D., Poisson J., Zhang Y. (2015). The I-TASSER Suite: Protein structure and function prediction. Nat. Methods.

[24] Geiger S.R., Lorenzen K., Schreieck A., Hanecker P., Kostrewa D., Heck A.J., Cramer P. (2010). RNA polymerase I contains a TFIIF-related DNA-binding subcomplex. Mol. Cell.

[25] Hoffmann N.A., Jakobi A.J., Moreno-Morcillo M., Glatt S., Kosinski J., Hagen W.J., Sachse C., Muller C.W. (2015). Molecular structures of unbound and transcribing RNA polymerase III. Nature.

[26] Gaiser F., Tan S., Richmond T.J. (2000). Novel dimerization fold of RAP30/RAP74 in human TFIIF at 1.7 A resolution. J. Mol. Biol..

[27] Freire-Picos M.A., Krishnamurthy S., Sun Z.W., Hampsey M. (2005). Evidence that the Tfg1/Tfg2 dimer interface of TFIIF lies near the active center of the RNA polymerase II initiation complex. Nucleic Acids Res..

[28] Penrod Y., Rothblum K., Rothblum L.I. (2012). Characterization of the interactions of mammalian RNA polymerase I associated proteins PAF53 and PAF49. Biochemistry.

[29] Schilbach S., Hantsche M., Tegunov D., Dienemann C., Wigge C., Urlaub H., Cramer P. (2017). Structures of transcription pre-initiation complex with TFIIH and Mediator. Nature.

[30] Conaway R.C., Garrett K.P., Hanley J.P., Conaway J.W. (1991). Mechanism of promoter selection by RNA polymerase II: Mammalian transcription factors alpha and beta gamma promote entry of polymerase into the preinitiation complex. Proc. Natl. Acad. Sci. USA.

[31] Tan S., Aso T., Conaway R.C., Conaway J.W. (1994). Roles for both the RAP30 and RAP74 subunits of transcription factor IIF in transcription initiation and elongation by RNA polymerase II. J. Biol. Chem..

[32] Ghazy M.A., Brodie S.A., Ammerman M.L., Ziegler L.M., Ponticelli A.S. (2004). Amino acid substitutions in yeast TFIIF confer upstream shifts in transcription initiation and altered interaction with RNA polymerase II. Mol. Cell Biol..

[33] Lei L., Ren D., Burton Z.F. (1999). The RAP74 subunit of human transcription factor IIF has similar roles in initiation and elongation. Mol. Cell Biol..

[34] Ren D., Lei L., Burton Z.F. (1999). A region within the RAP74 subunit of human transcription factor IIF is critical for initiation but dispensable for complex assembly. Mol. Cell Biol..

[35] Yan Q., Moreland R.J., Conaway J.W., Conaway R.C. (1999). Dual roles for transcription factor IIF in promoter escape by RNA polymerase II. J. Biol. Chem..

[36] Fishburn J., Hahn S. (2012). Architecture of the yeast RNA polymerase II open complex and regulation of activity by TFIIF. Mol. Cell Biol..

[37] Khaperskyy D.A., Ammerman M.L., Majovski R.C., Ponticelli A.S. (2008). Functions of Saccharomyces cerevisiae TFIIF during transcription start site utilization. Mol. Cell Biol..

[38] McNamar R., Abu-Adas Z., Rothblum K., Knutson B.A., Rothblum L.I. (2019). Conditional depletion of the RNA polymerase I subunit PAF53 reveals that it is essential for mitosis and enables identification of functional domains. J. Biol Chem..

[39] Sainsbury S., Bernecky C., Cramer P. (2015). Structural basis of transcription initiation by RNA polymerase II. Nat. Rev. Mol. Cell Biol..

[40] Engel C., Gubbey T., Neyer S., Sainsbury S., Oberthuer C., Baejen C., Bernecky C., Cramer P. (2017). Structural Basis of RNA Polymerase I Transcription Initiation. Cell.

[41] Tafur L., Sadian Y., Hoffmann N.A., Jakobi A.J., Wetzel R., Hagen W.J.H., Sachse C., Muller C.W. (2016). Molecular Structures of Transcribing RNA Polymerase I. Mol. Cell.

[42] Sadian Y., Tafur L., Kosinski J., Jakobi A.J., Wetzel R., Buczak K., Hagen W.J., Beck M., Sachse C., Muller C.W. (2017). Structural insights into transcription initiation by yeast RNA polymerase I. EMBO J..

[43] Fernandez-Tornero C., Moreno-Morcillo M., Rashid U.J., Taylor N.M., Ruiz F.M., Gruene T., Legrand P., Steuerwald U., Muller C.W. (2013). Crystal structure of the 14-subunit RNA polymerase I. Nature.

[44] Tafur L., Sadian Y., Hanske J., Wetzel R., Weis F., Muller C.W. (2019). The cryo-EM structure of a 12-subunit variant of RNA polymerase I reveals dissociation of the A49-A34.5 heterodimer and rearrangement of subunit A12.2. Elife.

[45] Liljelund P., Mariotte S., Buhler J.M., Sentenac A. (1992). Characterization and mutagenesis of the gene encoding the A49 subunit of RNA polymerase A in Saccharomyces cerevisiae. Proc. Natl. Acad. Sci. USA.

[46] Gadal O., Mariotte-Labarre S., Chedin S., Quemeneur E., Carles C., Sentenac A., Thuriaux P. (1997). A34.5, a nonessential component of yeast RNA polymerase I, cooperates with subunit A14 and DNA topoisomerase I to produce a functional rRNA synthesis machine. Mol. Cell Biol..

[47] Rothblum L.I., Rothblum K., Chang E. (2016). PAF53 is essential in mammalian cells: CRISPR/Cas9 fails to eliminate PAF53 expression. Gene.

[48] Natsume T., Kiyomitsu T., Saga Y., Kanemaki M.T. (2016). Rapid Protein Depletion in Human Cells by Auxin-Inducible Degron Tagging with Short Homology Donors. Cell Rep..

[49] Lin D.W., Chung B.P., Huang J.W., Wang X.R., Huang L., Kaiser P. (2019). Microhomology-based CRISPR tagging tools for protein tracking, purification, and depletion. J. Biol. Chem..

[50] Nakagawa K., Hisatake K., Imazawa Y., Ishiguro A., Matsumoto M., Pape L., Ishihama A., Nogi Y. (2003). The fission yeast RPA51 is a functional homolog of the budding yeast A49 subunit of RNA polymerase I and required for maximizing transcription of ribosomal DNA. Genes Genet. Syst..

[51] Jao C.Y., Salic A. (2008). Exploring RNA transcription and turnover in vivo by using click chemistry. Proc. Natl. Acad. Sci. USA.

[52] Peyroche G., Milkereit P., Bischler N., Tschochner H., Schultz P., Sentenac A., Carles C., Riva M. (2000). The recruitment of RNA polymerase I on rDNA is mediated by the interaction of the A43 subunit with Rrn3. EMBO J..

[53] Han Y., Yan C., Nguyen T.H.D., Jackobel A.J., Ivanov I., Knutson B.A., He Y. (2017). Structural mechanism of ATP-independent transcription initiation by RNA polymerase I. Elife.

[54] Sadian Y., Baudin F., Tafur L., Murciano B., Wetzel R., Weis F., Muller C.W. (2019). Molecular insight into RNA polymerase I promoter recognition and promoter melting. Nat. Commun..

[55] Schneider D.A. (2012). RNA polymerase I activity is regulated at multiple steps in the transcription cycle: Recent insights into factors that influence transcription elongation. Gene.

[56] Kuhn C.D., Geiger S.R., Baumli S., Gartmann M., Gerber J., Jennebach S., Mielke T., Tschochner H., Beckmann R., Cramer P. (2007). Functional architecture of RNA polymerase I. Cell.

[57] Mason S.W., Wallisch M., Grummt I. (1997). RNA polymerase I transcription termination: Similar mechanisms are employed by yeast and mammals. J. Mol. Biol..

[58] Nemeth A., Perez-Fernandez J., Merkl P., Hamperl S., Gerber J., Griesenbeck J., Tschochner H. (2013). RNA polymerase I termination: Where is the end?. Biochim. Biophys. Acta.

[59] Kuhn A., Grummt I. (1989). 3′-end formation of mouse pre-rRNA involves both transcription termination and a specific processing reaction. Genes Dev..

[60] Hannan R.D., Hempel W.M., Cavanaugh A., Arino T., Dimitrov S.I., Moss T., Rothblum L. (1998). Affinity purification of mammalian RNA polymerase I. Identification of an associated kinase. J. Biol. Chem..

[61] Seither P., Zatsepina O., Hoffmann M., Grummt I. (1997). Constitutive and strong association of PAF53 with RNA polymerase I. Chromosoma.

[62] Hannan K.M., Rothblum L.I., Jefferson L.S. (1998). Regulation of ribosomal DNA transcription by insulin. Am. J. Physiol..

[63] Darriere T., Pilsl M., Sarthou M.K., Chauvier A., Genty T., Audibert S., Dez C., Leger-Silvestre I., Normand C., Henras A.K. (2019). Genetic analyses led to the discovery of a super-active mutant of the RNA polymerase I. PLoS Genet..

[64] Wang T., Birsoy K., Hughes N.W., Krupczak K.M., Post Y., Wei J.J., Lander E.S., Sabatini D.M. (2015). Identification and characterization of essential genes in the human genome. Science.

[65] Wang T., Wei J.J., Sabatini D.M., Lander E.S. (2014). Genetic screens in human cells using the CRISPR-Cas9 system. Science.

[66] Shalem O., Sanjana N.E., Hartenian E., Shi X., Scott D.A., Mikkelsen T.S., Heckl D., Ebert B.L., Root D.E., Doench J.G. (2014). Genome-scale CRISPR-Cas9 knockout screening in human cells. Science.

[67] Panov K.I., Panova T.B., Gadal O., Nishiyama K., Saito T., Russell J., Zomerdijk J.C. (2006). RNA polymerase I-specific subunit CAST/hPAF49 has a role in the activation of transcription by upstream binding factor. Mol. Cell Biol..

[68] Choudhary C., Kumar C., Gnad F., Nielsen M.L., Rehman M., Walther T.C., Olsen J.V., Mann M. (2009). Lysine acetylation targets protein complexes and co-regulates major cellular functions. Science.

[69] Chen S., Seiler J., Santiago-Reichelt M., Felbel K., Grummt I., Voit R. (2013). Repression of RNA polymerase I upon stress is caused by inhibition of RNA-dependent deacetylation of PAF53 by SIRT7. Mol. Cell.

[70] Tsai Y.C., Greco T.M., Cristea I.M. (2014). Sirtuin 7 plays a role in ribosome biogenesis and protein synthesis. Mol. Cell Proteom..

[71] Grob A., Roussel P., Wright J.E., McStay B., Hernandez-Verdun D., Sirri V. (2009). Involvement of SIRT7 in resumption of rDNA transcription at the exit from mitosis. J. Cell Sci..

[72] Ford E., Voit R., Liszt G., Magin C., Grummt I., Guarente L. (2006). Mammalian Sir2 homolog SIRT7 is an activator of RNA polymerase I transcription. Genes Dev..

[73] Kiran S., Chatterjee N., Singh S., Kaul S.C., Wadhwa R., Ramakrishna G. (2013). Intracellular distribution of human SIRT7 and mapping of the nuclear/nucleolar localization signal. FEBS J..

